# European position paper on polypharmacy and fall-risk-increasing drugs recommendations in the World Guidelines for Falls Prevention and Management: implications and implementation

**DOI:** 10.1007/s41999-023-00824-8

**Published:** 2023-07-15

**Authors:** Nathalie van der Velde, Lotta J. Seppala, Sirpa Hartikainen, Nellie Kamkar, Louise Mallet, Tahir Masud, Manuel Montero-Odasso, Eveline P. van Poelgeest, Katja Thomsen, Jesper Ryg, Mirko Petrovic, The EuGMS Task, Finish Group on Fall-risk-increasing drugs

**Affiliations:** 1 Amsterdam UMC, Department of Internal Medicine, Section of Geriatric Medicine, University of Amsterdam, Amsterdam, The Netherlands; 2 Amsterdam Public Health Research Institute, Amsterdam, The Netherlands; 3 School of Pharmacy, University of Eastern Finland, Kuopio, Finland; 4 Gait and Brain Laboratory, Lawson Research Health Institute, Parkwood Hospital, London, ON Canada; 5 Faculty of Pharmacy, University of Montreal, Montréal, Québec Canada; 6 Nottingham University Hospitals NHS Trust, Nottingham, UK; 7 Gait and Brain Laboratory, Lawson Research Health Institute, Parkwood Hospital, London, ON Canada; 8 Department of Geriatric Medicine, Odense University Hospital, Odense, Denmark; 9 Department of Internal Medicine and Paediatrics, Section of Geriatrics, Faculty of Medicine and Health Sciences, Ghent University, Ghent, Belgium; 10 Department of Epidemiology and Biostatistics, University of Western Ontario, London, ON Canada; 11 Department of Pharmacy and Geriatrics, McGill University Health Center, Montréal, QC Canada; 12 Schulich School of Medicine and Dentistry, London, ON Canada; 13 Departments of Medicine (Geriatrics) and of Epidemiology and Biostatistics, University of Western Ontario, London, ON Canada; 14 Geriatric Research Unit, Department of Clinical Research, University of Southern Denmark, Odense, Denmark

**Keywords:** Falls prevention, Medication review, Deprescribing, Polypharmacy, Adverse drug reactions, Fall-risk-increasing drugs, Implementation

## Abstract

Falls prevention and management in older adults is a critical global challenge. One of the key risk factors for falls is the use of certain medications. Therefore, to prevent medication-related falls, the following is recommended in the recent World Guidelines for Falls Prevention and Management: (1) assess for fall history and the risk of falls before prescribing potential fall-risk-increasing drugs (FRIDs), (2) use a validated, structured screening and assessment tool to identify FRIDs when performing a medication review, (3) include medication review and appropriate deprescribing of FRIDs as a part of the multifactorial falls prevention intervention, and (4) in long-term care residents, if multifactorial intervention cannot be conducted due to limited resources, the falls prevention strategy should still always include deprescribing of FRIDs.

In the present statement paper, the working group on medication-related falls of the World Guidelines for Falls Prevention and Management, in collaboration with the European Geriatric Medicine Society (EuGMS) Task and Finish group on FRIDs, outlines its position on how to implement and execute these recommendations in clinical practice.

Preferably, the medication review should be conducted as part of a comprehensive geriatric assessment to produce a personalized and patient-centered assessment. Furthermore, the major pitfall of the published intervention studies so far is the suboptimal implementation of medication review and deprescribing. For the future, it is important to focus on gaining which elements determine successful implementation and apply the concepts of implementation science to decrease the gap between research and practice.

## Key summary points

**Aim:**

The recent World Guidelines for Falls Prevention and Management provide several recommendations on how to prevent medication-related falls.

**Findings:**

Medication review and deprescribing are key interventions in falls prevention and should be structured, personalized, and patient-centered. Preferably, the medication review should be conducted as part of a comprehensive geriatric assessment.

**Message:**

Improved information sharing between various prescribers, deprescribing recommendations implemented in guidelines, and increased education and training for health care professionals are warranted to facilitate the deprescribing process.

## Introduction

In light of the world’s ageing population, prevention and management of falls is a critical global challenge due to fall-related negative effects on functional independence, quality of life, morbidity, mortality, and health-related costs [1]. Numerous clinical practice guidelines for falls prevention have been published so far [2]. However, these guidelines are not uniform, and several gaps persist [2]. Therefore, in 2019, a group of experts started a global initiative to create a new set of globally applicable clinical practice guidelines for falls prevention and management [3]. The World Guidelines for Falls Prevention and Management (WFG) were published 2022 and include recommendations on fall risk stratification, details of assessment and intervention components and combinations, and recommendations for specific settings and populations [1]. According to the WFG, the core of falls prevention contains (i) risk assessment and stratification; (ii) general recommendations on optimising physical function and mobility for all and (iii) offering a holistic, multidomain intervention to older adults at high risk of falls. The detailed recommendations and evidence summaries can be found in the WFG. In this statement paper we discuss the recommendations (Box 1) and practical tips related to medication-related falls produced by the Polypharmacy and Fall-risk-increasing Drugs (FRIDs) Working group. In addition, in collaboration with the European Geriatric Medicine Society (EuGMS) Task and Finish group on FRIDs, we will outline how to implement and execute these recommendations in clinical practice and set the agenda for future research within the topic. Considering the warranted multidisciplinary approach for medication review and deprescribing, the discussed recommendations, practical tips and implementations considerations are relevant for all health care professionals engaging in falls and/or pharmacotherapy in older persons.


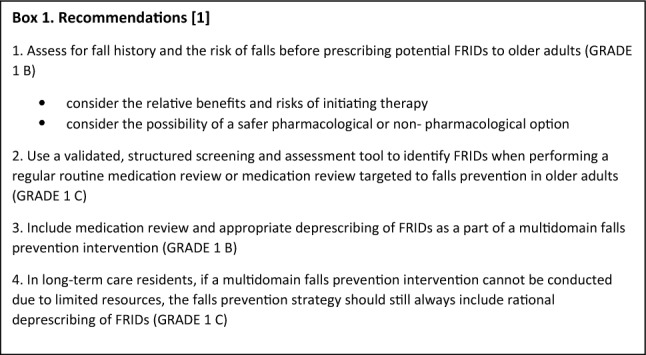


GRADE (the Grading of Recommendations Assessment, Development and Evaluation). GRADE informs whether the recommendation is strong (1) or weak (2) based on the quality of evidence being high (A), moderate (B), or low (C). [4]

## Fall-risk-increasing drugs

One of the objectives of the working group was to summarize the literature regarding FRIDs as risk factors for falling. The working group concluded, that based on currently existing systematic reviews and meta-analyses there is strong evidence that several medication classes including all types of psychotropics, antiepileptics, anticholinergics, and some classes of cardiovascular medications are FRIDs [1]. Particularly, psychotropics have been consistently associated with increased risk of falls across different meta-analyses [5–12]. However, published systematic reviews and meta-analyses have certain limitations, as some of the medication groups have not been studied extensively [13]. Furthermore, the focus of these studies is often the overall effect, and we know little about personalized risks [13]. Therefore, in the recently developed STOPPFall tool, a list of FRIDs was created based on literature and expert opinion [13]. The use of STOPPFall as a screening tool to identify FRIDs when performing a medication review in older adults with a fall history is endorsed by the EuGMS [13]. In Table 1, the 14 medication classes belonging to the STOPPFall consensus list are presented [13]. In addition, we have added the possible mechanisms leading to falls for each medication class in Table 1 based on literature. A more detailed description on differences between pharmacological subclasses with regard to fall-risk-increasing properties can be found in the STOPPFall article [13].

**Table 1:** Fall-risk increasing drugs according to STOPPFall [13] and their possible mechanisms leading to falls based on literature*

Alpha-blockers used as antihypertensives
Orthostatic hypotension, hypotension, dizziness, asthenia, blurred vision, sleep disorders, arrhythmia and syncope [14, 15]
Alpha-blockers used for prostate hyperplasia
Orthostatic hypotension, hypotension, dizziness, somnolence, visual impairment, and syncope [16, 17]
Anticholinergics
Effects on central nervous system (sedation, confusion, delirium, dizziness, cognitive impairment, impaired concentration) and blurred vision, tachycardia [18]
Antidepressants
Sedation, impaired balance/reaction time, orthostatic hypotension, cardiac conduction and rhythm disorders, visual impairment, hyponatremia, delirium, and drug-induced movement disorders [19]
Antiepileptics
Drowsiness, fatigue, dizziness, unsteadiness, vertigo, imbalance, diplopia, cognitive impairment, confusion, drug-induced movement disorders and hyponatraemia [20]
Antihistamines
Central nervous system side effects (e.g., sedation, drowsiness, somnolence, fatigue, cognitive decline), anticholinergic effects, cardiovascular toxicities (e.g., arrhytmias, prolongation of the QT interval, and postural hypotension) [21]
Antipsychotics
Sedation, drowsiness or somnolence, dizziness or vertigo, orthostatic hypotension, hypotension, drug-induced movement disorders, cardiac effects (QTc prolongation and tachycardia), anticholinergic effects (e.g., blurred vision), delirium, confusion, hyponatremia [22, 23]
Benzodiazepines and benzodiazepine-related drugs
Muscular weakness, ataxia, sedation, extrapyramidal symptoms, imbalance and/or dizziness, visual disorders, delirium, orthostatic hypotension [24]
Centrally acting antihypertensives
Orthostatic hypotension, hypotension, syncope, dizziness/vertigo, sleep disorders, arrhythmia, weakness, drowsiness and sedation [25] [15]
Diuretics
Orthostatic hypotension, hypotension, syncope, dizziness, sedation, electrolyte disturbances and volume depletion [26]
Opioids
Orthostatic hypotension, hypotension, drowsiness, somnolence, dizziness or vertigo, sedation, confusion, delirium, eye disorders, muscle problems (e.g., rigidity) [27]
Overactive bladder and incontinence medications
Dizziness or vertigo, somnolence, delirium, visual impairment [17, 28]
Vasodilators used in cardiac diseases
Hypotension, orthostatic hypotension, dizziness, rhythm disorders, somnolence and syncope [29]

*The mechanisms listed in the table are based on clinical reviews produced by the EuGMS Task and Finish Group regarding therapeutic dilemmas related to FRIDs and associated conditions and other overview articles

## Before considering prescribing fall-risk-increasing drugs

There is strong evidence that certain medications can increase fall risk in older adults. Therefore, the first recommendation by the working group is to assess for fall history and the risk of falls *before* prescribing FRIDs to older adults [1]. An eight-step approach has been proposed for appropriate prescribing by Pollock et al. (as pasted below) and additional details regarding FRIDs prescribing have been added [1, 30]:

1. Assess and clearly define the patient’s problem
  Any new symptom in an older adult should be considered as a possible side effect to avoid prescribing cascades. New side effects can develop even if the patient has taken the medication for a long time (e.g., due to changes in pharmacokinetics and pharmacodynamics).
2. Specify the therapeutic objective
  Define treatment goals together with the patient
3. Select the appropriate drug therapy and consider:
  The relative benefits and risks of intended therapy.
  Patient’s characteristics, co-morbidities including geriatric syndromes such as falls, frailty or cognitive impairment, renal function, polypharmacy, other FRIDs, life expectancy, and patient’s preferences.
  Shared-decision making as it has been associated with better-informed patients and improved patient compliance.
  Minimum effective dosage and shortest prescription period if this personalized strategy leads to the conclusion that FRIDs prescription is necessary.
  Define timeframe for drug therapy evaluation.
4. Initiate therapy with specific prescription details and consider non-pharmacologic alternatives
5. Provide information, instructions, and warnings
6. Assess therapy regularly (e.g., monitor treatment results and consider discontinuation of the drug)
7. Monitoring should be performed to identify if patients are developing adverse drug events, such as falls or cognitive, motoric or sensory impairment
8. Consider drug costs for patients and society when prescribing
9. Use clinical decision support systems and other tools to reduce prescribing errors
  There are numerous explicit tools, which can be used to guide appropriate prescribing including STOPP/START, STOPPFall, STOPPFrail, Beers criteria, FORTA, TIME, REMEDI[e]S, and Web-based Meds75 + guide [13, 31, 32, 38].

## Regular medication review

Due to dynamic health conditions in older people and potential polypharmacy-related harm, regular medication review is important [39]. The changes in risk versus benefit ratios of medications over time underline the need for regular reassessments. Moreover, the current health care systems are highly complex with multiple prescribers leading to an increased risk of potential medication errors and adverse drug events, which can be counterbalanced by regular medication reviews. As for fall risk, the exposure to FRIDs should be kept as short as possible (i.e., only if clinically indicated, with the help of regular medication review). Older adults living with frailty are particularly prone to rapid changes in their health condition and, therefore, at increased risk of falls and adverse drug events. Thus, in older adults living with frailty or cognitive impairment, medication review (including FRIDs review) should preferably be performed at least every 6 months [40]. In non-frail older adults, medication review (including FRIDs review) should preferably be performed at least annually [41]. In addition, medication review should be conducted at every acute change of health status.

## Medication review and deprescribing as part of multidomain falls preventive intervention or as a single intervention

Multidomain falls preventive interventions (i.e., a combination of interventions tailored to the individual), when followed and delivered, are effective and are, therefore, a key element of falls prevention [1]. One of the aims of the working group was to ascertain whether a medication review should be included in this multifactorial strategy. In a recent network meta-analysis, a basic fall-risk assessment (including medication review) was one of the effective components in addition to physical exercise, assistive technology, environmental assessment and modifications, and quality improvement strategies that were associated with reductions in number of fallers and falls rate [42]. In addition, a systematic review of published falls prevention guidelines from 2021 reported that the majority of guidelines included a medication review as one of the components of the multifactorial strategy [2]. Therefore, the working group recommends that a medication review and appropriate deprescribing of FRIDs should be a standard component of multifactorial intervention [1].

The working group also conducted a systematic review and meta-analysis to investigate whether medication review is an effective single intervention for falls prevention [43]. No significant associations between medication reviews in community dwellers, long-term care residents, or hospitalized patients and fall outcomes were found [43]. However, in the meta-analysis assessing medication reviews in long-term care, there was a trend for a lower number of fallers (risk ratio 0.86; 95% confidence interval 0.72–1.02) [43]. Long-term care residents are generally frail and at increased risk of adverse drug events. Among them, medication review might already be effective, as a single intervention [43]. However, considering the multifactorial nature of falls, single interventions are neither appropriate in falls prevention in general, nor in this frail population [1]. Therefore, the working group conditionally recommends that in long-term care residents, if a multidomain intervention cannot be conducted due to limited resources, the falls prevention strategy should still always include deprescribing of FRIDs [1]. It is very important that each fall is carefully registered in nursing homes, so that medication review can always be performed after a fall in a post-fall assessment. The importance of adequate registration is equally valid for hospital setting.

## How to perform a fall-risk-increasing drugs medication review

Finally, the working group summarized how medication review and deprescribing should be conducted. The working group included the use of validated, structured screening and assessment tool to identify FRIDs when performing a medication review and applying deprescribing in the recommendations [1]. This is in line with the existing guidelines on medication review, such as the English National Institute for Health and Care Excellence (NICE), which state that medication review should be structured [44]. To assist a structured approach, the five steps of patient-centered deprescribing [i.e., (1) comprehensive medication history, (2) Identify potentially inappropriate medications, (3) determine if medications can be prioritized for deprescribing, (4) plan and initiate withdrawal and (5) monitoring support and documentation] are listed below [45, 46]. Preferably, the medication review and deprescribing should be conducted as part of a comprehensive geriatric assessment as explained in more detail in step 3. Furthermore, multidisciplinary approach is very important when performing medication review and deprescribing. Professionals in addition physicians and pharmacists, such as nurses, physiotherapists, occupational therapists, and nutritionists, play also an important role in this multidisciplinary approach. The exact roles of different professionals (e.g., physicians, pharmacists) in undertaking medication review and deprescribing and different steps is determined by the health care structure of a specific country.

### Comprehensive medication history

In a comprehensive medication history, a complete list of all prescription and over the counter medications should be collected including medications that are used regularly, on demand, and intermittently [45]. In addition, the non-medical use of prescription drugs should be addressed. In addition, information on use of vitamins or natural products as well as on products that have been ordered online should also be included. For each medication, the dose, frequency, formulation, administration route, administration time, duration of use, and patient-reported indication should be collected. Furthermore, information on how the patient takes the medication should be included (e.g., including whether somebody assists the patient to take the medication or whether tools such as dosage boxes are being used and whether they take the medication while fasting or not). It is important to assess how often the patients redeem their prescriptions to account for adherence problems or to assess whether the patient is taking too many pills. In addition, any previous allergies, intolerances, or experienced adverse drug reactions (ADRs, i.e., responses to a drug which is noxious and unintended and occurring at doses used in humans for prophylaxis, diagnosis or therapy of diseases, or for the modification of physiological function) and adverse drug events (ADEs, i.e., any injuries resulting from medical interventions related to a drug. This includes both adverse drug reactions in which no error occurred and complications resulting from medication errors) should also be collected [45]. During the comprehensive medication history, the patients can report which medications they associate with ADRs or ADEs, which ones they experience as not needed, and which ones they value [45].

### Identify potentially inappropriate medications

Medication review and deprescribing should be personalized due to heterogeneity among older adults. Within this personalized strategy, the potential beneficial effects of therapy must be balanced against potential and experienced adverse drug events [47]. Medication review and deprescribing is often a challenging process, frequently resulting in therapeutic dilemmas [19, 27].

Illustrative examples are depression and antidepressants, or pain and opioids. Both the conditions and medications to treat those conditions have been linked to increased fall risk [19, 27]. The EuGMS Task and Finish Group is currently producing a series of clinical reviews on these therapeutic dilemmas [19, 27]. These can assist the clinician in coping with these dilemmas in clinical practice [19, 27]. Furthermore, earlier studies have found that patients’ characteristics play a role in the effect of FRIDs on fall risk [48]. For example, frailty can lead to amplified adverse effects of drugs due to increased vulnerability to stressors; or cognitive or sensory impairment can result in mistakes in taking medications [39]. Thus, characteristics, such as co-morbidities including geriatric syndromes like falls, frailty or cognitive impairment, other FRIDs, polypharmacy, life expectancy, and patients’ preferences should be taken into account when conducting medication reviews and deprescribing [45].

There is a high degree of variability in older patients’ individual care goals, values, and preferences. This was demonstrated in a study by Tinetti et al. in which half of patients ranked prevention of cardiovascular events as more important than reducing the risk of the combination of fall injuries and medication symptoms [49]. The other half ranked the health goals in the opposite order [49]. Fried et al., reported that around three quarters of the older adults ranked maintaining independence as the most important health goal over staying alive, pain, and symptom relief [50]. In contrast, disease specific-guideline recommendations are usually based on evidence from randomized controlled trials (RCTs) investigating “hard endpoints”, such as cardiovascular events or mortality. Furthermore, the level of evidence on the benefit versus risk ratio of medications is low in older adults, as the supporting evidence is established usually via RCTs in relatively young patients with a single disease. In general, prioritization of care goals can be used as a starting point for discussing what matters most to older adults with multimorbidity [50].

In summary, the medication review will thus lead to varying recommendations in different individuals due to the heterogeneity in older adults’ characteristics, their respective pharmacotherapy, and their unique goals and wishes. Preferably, the medication review is conducted as a part of comprehensive geriatric assessment, as the latter process takes all relevant information into account in a structured and holistic way.

Furthermore, an appropriate tool that is easy to use can help in identifying inappropriate drugs. For falls prevention, a practical method to identify and review FRIDs is to employ the STOPPFall tool derived via international consensus, which is freely available online [13].

### Determine if medications can be prioritized for deprescribing

The decision on possible deprescribing attempts should be further based on patient consent, appropriate timing of deprescribing, previous deprescribing attempts, and reasons for possible failures [45]. In general, geriatric patients and nursing home residents are open to deprescribing, especially if this is proposed by their treating physician [51]. Reeve et al. listed that the following items should be included in a personalized discussion with the patient to optimize the patient’s willingness for attempting deprescribing [45]:

1. Introduce deprescribing in a manner that does not evoke stress nor fear or impair the patient–prescriber relationship
2. Emphasize that deprescribing is recommended to achieve health goals and not, because the patient is ‘not worth treating’
3. Emphasize potential benefits of deprescribing to patient when discussing lack of benefits/necessity of the medication and the potential risks associated with its use, such as falls
4. Discuss what will be done to minimize the risks of deprescribing, and confirmation that deprescribing is an attempt

### Plan and initiate withdrawal

How to initiate deprescribing needs to be decided before a deprescribing attempt can be started [45]. It should be determined whether the medication needs to be withdrawn in a stepwise manner and how this should be performed [13, 45].

### Monitoring support and documentation

It is important to aim for sustainable deprescribing interventions. The current available data suggest that the rates of complete discontinuation of at least one FRID ranges from 10 to 40% [52] depending on medication class and on the original indication for the FRID [53]. Re-prescribing can be indicated if symptoms re-occur after stopping the drug [45]. It is important to differentiate the symptoms from adverse drug withdrawal reactions. The long-term success of deprescribing can be maximized by monitoring, support, and documentation [45]. Feeling supported is highly valued by patients during the deprescribing process [45]. To minimize unnecessary re-initiation of deprescribed medications, it is important to communicate deprescribing decisions and the process undertaken that led to the deprescribing decision between different prescribers [45].

### Implementation and future research

Successful implementation strategies for medication review and deprescribing are warranted. In current clinical practice, regrettably, there is reluctance towards deprescribing due to its complexity. This was also shown in the systematic review by the working group, as often healthcare providers did not change medications based on the recommendations given by, for example, a pharmacist or a clinical decision support system [43].

Furthermore, two recently published large-scale clinical trials on medication reviews (i.e., SENATOR and OPERAM using STOPP/START version 2) as the key component of the interventions tested, were not effective in terms of key endpoints; this was most likely due to unsuccessful implementation of STOPP/START criteria by prescribers [54, 55]. In general, the barriers and enablers for deprescribing have been classified into environmental (e.g., regulatory, financial, policy), healthcare organization, provider, and patient/public-related factors [56].

According to a recent European survey among geriatricians and geriatricians-in-training, the most important barriers for deprescribing were patients’ unwillingness, fear of negative consequences, lack of time, and poor communication between multiple prescribers [57]. The respondents stated that their future deprescribing activities would probably increase with improved information sharing between various prescribers, deprescribing recommendations in guidelines, and increased education and training [57].

It is thus evident that future implementation strategies for effective deprescribing should be multifaceted and target several levels of health care systems. As medication review and deprescribing are standard components of the multidomain falls prevention intervention, the seven steps that have been described as part of the falls prevention implementation plan are listed in box 2 [58]. Such an implementation plan should be tailored to local context and needs.


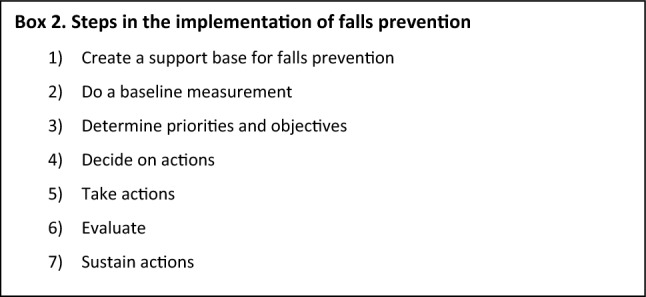


Furthermore, referral processes should be simple for individuals with very high risk of medication-related falls, as there is a loss of older persons during each step of the referral processes (screening–assessment–referral–intervention) [59, 60].

To date, there has been limited adaption of concepts from implementation science in medication review and deprescribing research and even less in clinical practice [61]. Understanding how to better conceptualize and evaluate successful medication review and deprescribing processes by considering implementation science could potentially increase the translation of the research into practice [61]. Ailabouni et al., suggested to (1) define, differentiate, and apply the concepts of “evidence-based intervention” and “implementation strategy” to the field of deprescribing, (2) understand the importance of context and describing contextual determinants through use of an implementation science framework, and (3) specify and evaluate deprescribing implementation outcomes according to existing typologies, to leverage and benefit from implementation science [61].

Finally, to further allow individualized prescribing plans, research on personalized treatment effects is warranted. To this end, high-quality studies should be conducted. The EuGMS Task and Finish group has described the items to increase the quality of the observational studies in their previous position paper [46]. Besides observational studies, falls and fall-related injuries should be included in the list of adverse events to be evaluated in future clinical trials. Modern data-driven methods may help us in the estimation of heterogeneous treatment effects and identification of the patient groups (e.g., based on patient characteristics such as frailty or cognitive decline or biomarkers for whom the treatment generates increased fall risk). Furthermore, studies on specific drugs in each FRIDs group is warranted to give insights on the specific risks related to each drug. In addition, the relationships between FRIDs use and fall risk are highly influenced by interacting factors, such as co-medication, comorbidities, and frailty. Potentially, a conceptual model of relevant mechanisms and their interactions (causal loop diagram) could be beneficial [62]. From the causal loop diagram, we could identify possible variables to intervene. Eventually, the number of possible interventions could be simulated and later tested experimentally.

## Conclusion

In summary, the World Guidelines for Falls Prevention and Management Working Group on Polypharmacy and FRIDs, in collaboration with the EuGMS Task and Finish group on FRIDs, advocate the following aspects to improve the prevention and management of medication-related falls:

1. The relative benefits and risks of initiating FRIDs should be assessed when considering prescribing FRIDs.
2. Medication review is a standard component of the multidomain falls prevention intervention.
3. Medication review and deprescribing should be structured, personalized and patient-centered. Preferably, the medication review should be conducted as part of a comprehensive geriatric assessment.
4. A structured medication review tool such as STOPPFall should preferably be used to identify and deprescribe FRIDs.
5. Suboptimal implementation of medication review and deprescribing is ineffective. Therefore,
  - The interventions should be developed in partnership with older adults and end-users to increase their uptake and adherence. In addition, the context in which the interventions are to be integrated should be considered.
  - For clinical practice, a locally adapted implementation plan for falls prevention (including medication review as a component of multidomain intervention) is warranted.
  - In terms of research, focus is needed on understanding the most successful approaches for implementation and leverage from implementation science to decrease the gap between research and practice.
6. To allow individualized prescribing plans, research on personalized treatment effects is warranted.
